# Dr. John Charles Steele

**Published:** 1892-11-12

**Authors:** 


### JOHN CHARLES STEELE, M.D., L.R.C.S. EDINBURGH, F.F.P.S. GLASGOW.

It is with deep regret that we have to report the death of Dr. Steele, so long and so well-known as the superintendent of Guy's Hospital. He died quite suddenly on Sunday when under the influence of an anæsthetic which was being administered by his own consent, in order to enable the operation of colotomy to be performed. Dr. Steele died immediately after the operation commenced. Formerly medical superintendent at the Glasgow Royal Infirmary he relinquished that appointment in order to become Superintendent at Guy's Hospital in 1853. For nearly forty years Dr. Steele has had responsible charge of the administration at Guy's, and during this long period has seen many changes and passed through several severe crises. He died in harness, leaving a record so honourable that it falls to the lot of few men to equal much less to surpass it. He was well and widely known to all who take an interest in the welfare of hospitals and kindred institutions. Ever ready to do service in the cause of a friend, his kind-heartedness, his geniality, his shrewd common sense and enduring perseverance made him a power for good in the hospital world.

Only a fortnight ago he came to us full of kindly projects for the benefit of others. In the course of a holiday just taken in Scotland, he had visited again all the scenes of his youth, and his account of his experiences and of the changes which had taken place there during the last half-century were most entertaining. Dr. Steele had many friends, and we are only expressing their sentiments when we state that he was a man so sympathetic and so kind as to endear himself to one and all alike. On not a few occasions during the last twenty-five years it has fallen to our lot to stand side by side with him when the strife was severe and the prospect doubtful, but never once did he fail, while resolutely adhering to principle, to endeavour to find not only a wise solution but one which would commend itself to all parties, and so cause a cessation of conflict to the no small advantage of everyone concerned. Essentially a peacemaker, Dr. Steele could be firmly severe when severity was warranted by the circumstances. We believe there will be a unanimous consensus of opinion in support of the views we have here expressed. He had so far as we know but one *bête noir*, and that was the American visitor who wanted to see the whole of Guy's Hospital "right off," and who had allowed himself about a quarter of an hour to do it in. His view was that the majority of Americans pay too little regard to the earnest side of work. Had he visited America and so afforded our American cousins an opportunity of returning the courtesies he had extended during many years to visitors at Guy's Hospital, we are confident that he would have moderated his views. As it was he held to the day of his death, that before leaving the United States it was desirable that those who came to Guy's should go through a course of steadying self-restraint, so as to fit them to derive the maximum of good from their visits to the representative institutions of the old country.

Dr. Steele was an able and fluent writer. Several of his papers will live in hospital literature. Amongst them, the Howard prize essay on "The Mortality in Hospitals," 1877; "Hospital Dietaries," 1873; and the "Charitable Aspects of Medical Relief," 1891. He contributed not a few papers to our columns, and, as a reviewer, his Scotch wit enabled him to criticise closely, but fairly, and so to make a lasting impression on the reader.

He took the warmest interest in the Royal National Pension Fund for Nurses from the date of its first inception, and he has been actively engaged as one of the representatives of the Nurse Training Schools in considering the true bearings of the proposals to establish nurse registration. The conclusions embodied in his report as chairman of the Special Committee of The Hospitals Association entitled, "An Enquiry into Nurse Registration," are sound and convincing. Although this report was written nearly five years ago, it is still the ablest monograph which has appeared on the subject. The nurses had no better friend than Dr. Steele, and his death will cause a feeling of sorrow amongst hundreds of nurses, matrons, and hospital officials throughout the length and breadth of the land.

Personally we have lost a dear friend and colleague who will be difficult to replace. But there is consolation in the thought that he has gone to his long home full of years, after accomplishing much good work. Truly may we say of Dr. Steele, "he rests from his labours, and his works do follow him."

The greater part of the funeral service was held in Guy's Hospital Chapel on Thursday last, and was attended by a host of sorrowing friends. Amongst them were most of the best known workers in the hospitals and medical institutions.

